# Characterization of Sweet Almond Oil Content of Four European Cultivars (*Ferragnes*, *Ferraduel*, *Fournat*, and *Marcona*) Recently Introduced in Morocco

**DOI:** 10.1155/2021/9141695

**Published:** 2021-08-30

**Authors:** Reda Melhaoui, Souhayla Kodad, Nadia Houmy, Kamal Belhaj, Farid Mansouri, Malika Abid, Mohamed Addi, Aatika Mihamou, Marianne Sindic, Hana Serghini-Caid, Ahmed Elamrani

**Affiliations:** ^1^Laboratory for Agricultural Productions Improvement, Biotechnology and Environment (LAPABE), Faculty of Sciences, University Mohammed First, BP-717, 60000 Oujda, Morocco; ^2^Analysis Quality and Risk Unit, Laboratory of Food Quality and Safety (QSPA), Gembloux Agro-Bio Tech, University of Liège, 5030 Gembloux, Belgium

## Abstract

This study concerns the characterization of oil content and quality indices for almond cultivars (*Marcona* (Mr), *Fournat* (Fn), *Ferragnes* (Fg), and *Ferraduel* (Fd)) recently introduced into marginal agricultural areas in eastern Morocco. These verities are known for their rusticity and late flowering stage. The analyzed almond oils showed low acidity and peroxide values ranging, respectively, from 0.32 to 0.36% and 1.88 to 3.18 meq O_2_/kg. Fatty acid (FA) profile revealed a predominance of the unsaturated FA represented essentially by the oleic (56.64–64.03%) and linoleic FA (24.57–29.80%). Triacylglycerol (TAG) analysis allowed the determination of eleven species with a remarkable dominance of trioleylglycerol (OOO: 30%) and dioleyllinoleoylglcerol (OOL: 27.25%). Regarding the minor compounds, the results showed that the total phenol content ranges between 85.33 and 141.66 mg/kg. Concerning the tocopherol content, the studied oils showed richness in these secondary metabolites (408.99–491.77 mg/kg) with a dominance of *α*-tocopherol. In comparison to their homologues in the Mediterranean area, the evaluated almond oils demonstrated a slight superiority in terms of quality, in particular, to those produced in Spain.

## 1. Introduction

Almond trees (*Prunus amygdalus dulcis*) are native species to Western Asia, in particular, Iran and the surrounding countries. Currently, almond trees are widely cultivated elsewhere. Due to its rusticity and its adaptation to drought/arid climate, almond tree becomes the main nut tree in the Mediterranean zones. Commonly planted varieties are the result of a combination of human and natural selection [1]. The USA is the world's largest producer of almonds, with an estimated average production of 1,872,500 tons in 2019, followed by Spain (339,033 tons) and Iran (139, 029 tons). Morocco is the fourth largest producer of almond nuts with an average production of 116,900 tons in 2018 [2].

Various studies have shown that the consumption of nuts has a positive effect against many pathologies such as hypertension, obesity, and metabolic syndrome [3, 4]. These positive effects of almond kernels are related to its composition which is rich in different sources of nutrients and health-promoting compounds [5]. Moreover, they represent a high amount of protein ranging from 20% to 25%, with the PDCAAS (Protein Digestibility Corrected Amino Acid Score) value for raw almonds ranging between 44.3 and 47.8% [6]. The oil presents the major component in the kernel ranging from 40% to 67% [7].The applications of this oil vary from the food and cosmetic sector to the complementary medicine, thanks to several health benefits, including anti-inflammatory, antihepatotoxicity, immunity-boosting, and modulatory effects on the inflammation [8, 9]. Moreover, the almond oil displays a rich lipid profile, in monounsaturated (63.42–78.03%) and polyunsaturated (14.41–27.01%) fatty acids [10, 11]. It also presents a good dietary source of antioxidants, such as tocopherols, polyphenols, and flavonoids [12, 13], while these minor components are characterized by high variability, especially for the antioxidants, such as polyphenols which vary between 36 mg/kg [14] and 760 mg/kg [15] and also the *α*-tocopherols dominant in almond oils which are preferentially utilized by the human body. This isoform of vitamin E in almond ranges from 85 mg/kg to 650 mg/kg [16]. These different concentrations of minor components could be due to crop year effect, agricultural practices [13], different types of extraction [17], or also to different treatment steps of almond before the extraction such as roasting [18]. Generally, tocopherols are more abundant than tocotrienols, which are only found in some plant species' fruits and seeds [19].

For the sustainable development of the almond sector in eastern Morocco, the Green Moroccan Program and the Belgium Development Agency cooperated for extra planting of 6000 ha of four European introduced varieties: *Fournat de Breznaud* (Fn), *Ferragnes* (Fg), and *Ferraduel* (Fd) French native and *Marcona* (Mr) Spain native. These cultivars have been chosen for their late flowering in March permitting to escape to the adverse effects of January frosts that characterize this region [20, 21]. In addition, this species diversification increases the genetic richness in our agricultural systems, allows a better resistance to diseases, and reduces the problems of farmers in the face of climate-related risks. The new varieties coming from different environments should be properly tested and assessed for their adaptability to our environmental conditions and our semiarid climate. As a result, the goal of this work is to characterize and evaluate the physicochemical parameters of almond oils obtained from four European varieties newly introduced in the eastern provinces of Morocco. Oxidative stability index, fatty acid triacylglycerol profiles, and tocopherol contents are the three main parameters that have been used in this study to evaluate the quality of almond oils. Moreover, the results of this study provide scientific information on the adaptability of these varieties in eastern Morocco and improve local production, recommending different botanical varieties that have a better adaptation than those currently used. Of course, the evaluation of the performance of these trees cannot be performed on the basis of one-year work; this is why our team has been following the evolution of different parameters for the last 5 years [22–24].

## 2. Materials and Methods

### 2.1. Almond Samples

The almond kernels were obtained in collaboration with the Economic Interest Group “SIDI BOUHRIA” in eastern Morocco (34°44′13.6″ N, 002°20′15.0″ W). Five kilograms of almonds of each variety (with shell) subdivided into three groups were harvested in the last phase of maturity (August) in two orchards (extensive mode of plantation). For trituration, we used an oil screw press, the speed was 70 RPM with a temperature of 100 C°, and the fine particles were removed by centrifugation at 3000 RPM for 15 min. Finally, the obtained almond oil samples were stored for further analysis at a temperature of 4 °C. Four main varieties grown in the studied region were analyzed, *Ferragnes* (Fg), *Ferraduel* (Fd), *Fournat de Breznaud* (Fn), and *Marcona* (Mr). The cultivation of almond trees is essentially rain-fed, along with additional irrigation provided to almond trees during April and June. This zone is characterized by an average annual precipitation ranging between 100 mm and 300 mm, with annual temperatures varying from a minimum of −2°C to a maximum of 43°C.

### 2.2. Analytical Methods

#### 2.2.1. Physicochemical Parameters and Oxidative Stability Index

Peroxide value (meq O_2_/kg) and free acidity (% oleic acid) were determined according to the official European methods [25] for olive oil. Oxidative stability index of almond oils was determined by the Rancimat method measured with a Metrohm model 743.3 ± 0.01 g of oils warmed at 100 ± 1.6 C° and an air flow of 20 L/h. The oxidative stability index of oils was expressed by the induction period (time necessary to achieve the inflection point of the conductivity curve).

#### 2.2.2. Extraction and Colorimetric Determination of Phenol Contents

The extraction and measurement methods used were described by Ollivier et al. [26] and Mansouri et al. [27]. 5 g of almond oil was added to 5 mL of a methanol/water solution (80/20 v/v); after 10 min of mixing the small vials of liquids in a quickly oscillating circular motion using a vortex, the mixture oil-methanol was centrifuged at 3800 rpm for 15 min. This operation was repeated twice with the methanolic phase recuperated. The phenolic contents were determined according to the Folin–Ciocalteu method, using caffeic acid as a standard by absorbance at 750 nm.

#### 2.2.3. Total Tocopherol Analysis

Tocopherol profile (*α*-, *β*-, and *γ*-tocopherols) was analyzed by HPLC with a fluorescence detector HPLC-FLD (Agilent Technologies series 1200 system, Agilent Technologies), equipped with an automatic injector, according to the AOCS method Ce 8–89 [28]. The separation of tocopherols has been carried out on an Uptisphere 120A° NH2 column (150 mm *∗* 3 mm, 3 *μ*m), Interchim (Montluçon, France), maintained at 30°C. The injection volume was 10 *μ*L. The mobile phase was hexane/2-propanol (99 : 1, v/v) eluted in isocratic conditions at a flow rate of 1 mL·min^−1^. The tocopherols were identified and quantified by external standardization.

#### 2.2.4. Analysis of Triglycerides

To determine the composition of triglycerides, almond oil was dissolved in acetone (9%) and filtered through 0.45 *μ*m membranes. Triglyceride profile was determined by high-performance liquid chromatography (HPLC) coupled with a refractive index detector. The column used is a type C18 reversed-phase column (ODS C18 : 250 × 5 mm, 5 *μ*m), and the mobile phase consisted of acetone/acetonitrile (60%/40% v : v). Elution was carried out in isocratic conditions at 1 ml/min. The sample separations were performed at ambient temperature. The elution order was a function of the number of carbon atoms and the degree of unsaturation. Triglyceride peaks were identified by comparison with known oils (olive oil and soybean oil) and the most important triglyceride standards OOO purchased from Sigma and by determining their equivalent carbon number (ECN).

#### 2.2.5. Fatty Acid Analysis

Fatty acid profile of almond oils was identified by a standard composed of 37 methyl esters of fatty acids. Before analysis, fatty acids were converted into fatty acid methyl esters; after adding 8 mL of hexane, the mixture was subjected to gas-chromatograph analysis using an HP 6890 series gas-chromatography system equipped with an FID detector and a capillary column (Supelco Omega wax: 30 m × 0.25 mm × 0.25 *μ*m). The injection volume was 1 *μ*l in the split-less mode. This method of analysis was described in [29].

#### 2.2.6. Statistical Analysis

Statistical analyses were performed with the SPSS software for Windows (SPSS.21, USA). The results were expressed as the mean values ± standard deviation. The normal distribution was evaluated by the Shapiro–Wilk test. One-Way ANOVA and Tukey test were used for the sample mean comparison, which was considered different at a 5% significance level. A linear correlation was determined by using the Pearson bivariate test between oxidative stability and oil chemical characteristics. Principal component analysis (PCA) was performed on the dataset in order to differentiate the almond oil varieties according to their composition and physicochemical properties.

## 3. Results and Discussion

### 3.1. Almond Oil Physicochemical Indexes

The International Organization for Standardization (ISO) and Codex Alimentarius are the most significant standardization organizations in the world when it comes to edible oils and fats (which issue standards covering a wide range of activities). These international standards are crucial for protecting consumer health and safety. Standardization allows all parties participating in the edible fat and oil sector to communicate in the same language. In this regard, Codex Alimentarius recommended the values less than 5% for acidity and 15 milliequivalents of active oxygen/kg oil for unrefined oils [30].

Free acidity (AF) and peroxide value (IP) are the first main parameters that define the physicochemical quality of almond oils.  Table 1 shows low values for the two parameters (AF and IP), indicating a good quality of almond oils for the 4 studied varieties. Free acidity determines the free fatty acid quantity, expressed by oleic acid. Lower acidity values indicate a good oil quality and the absence or inexistence of enzymatic hydrolysis of acylglycerols [31]. The difference in AF values was not significant between the studied varieties (*p* > 0.05), in which the values ranged from 0.32% to 0.36%, not exceeding the recommended 5% by Codex Alimentarius for unrefined oils. The peroxide index is an indicator of the primary oxidation products (hydroperoxides). The oils which have high values of this later are presenting potential problems of secondary oxidation products such as ketones, aldehydes, short hydrocarbon chains, and alcohols [32]. In this study, significant differences have been observed for the peroxide index (*p* < 0.05), the lowest value was registered for the *Marcona* variety (1.88 meq O_2_ kg^−1^), while the highest value was found in the *Ferragnes* variety (3.18 meq O_2_ kg^−1^). All the obtained values are under the Codex Alimentarius recommendation for not exceeding 15 meq O_2_ kg^−1^ [33].

### 3.2. Total Phenol Content of Almond Oils

Phenolic compounds are natural secondary metabolites that present many biological effects, where the antioxidant capacity is the most important characteristic mainly for its beneficial impact on health [34]. Most of the phenol compounds are localized in almond kernels presenting a high variation of total phenol content, which varied from a minimum of 237 mg GAE/kg to a maximum of 12541.2 mg·GAE/kg [35–37]. Generally, this variation depends on several factors, such as variety, oil extraction method, and agricultural practices. The concentrations of total phenolic content in the studied almond oils range between 85.33 mg/kg for the Fn variety and 141.66 mg/kg for the Mr variety. The low value of total phenolic content observed between the compared almond kernels oils was due to the reduced solubility of phenolic compounds in the lipid fraction and at the same time, due to the high phenolic content present in the almond skin [11, 38], which was eliminated by the mechanical oil extraction. The significant differences between the values of our results mentioned in Table 2 are probably due to the effect of variety. Other studies by Rabadan et al. [39] have shown a less value of phenol content ranged from 18.53 mg/kg to 28.07 mg/kg for *Marcona*, *Ferragnes*, and *Ferraduel*.

### 3.3. Tocopherol Contents

Tocopherols are presented by four homologs (*α*-, *β*-, *γ*-, and *δ*-tocopherols), as determined by the position and the number of methyl groups on the chromanol ring [40]. Tocopherol compounds contribute to the antioxidant properties of oils; they also have protective roles in biological systems, as well as other neuroprotective properties. In addition to the abovementioned activities as an antioxidant, Vitamin E is involved in immunological function, anti-inflammatory processes, platelet aggregation inhibition, cell signaling, gene expression control, and other metabolic processes, in addition to the antioxidant functions [41]. Almond oil profile analysis showed the presence of three tocopherol homologs (*α*-, *β*-, and, *γ*-tocopherol) with the *α*-tocopherol as a dominant compound in the four studied varieties. Tocopherol compositions in almond oil are summarized in Table 2. The average of total tocopherols ranged from 408.99 mg/kg to 491.76 mg/kg, with *α*-tocopherol varying between 401.23 mg/kg and 473.55 mg/kg for Fg and Fn, respectively, while *γ*-tocopherol varies from 2.29 mg/kg to 14.77 mg/kg. *β*-tocopherol has the lower homolog content with an average ranging from 1.67 mg/kg to 3.43 mg/kg for Mr and Fn, respectively. Thus, these three homologs of tocopherols were found in similar studies with also a dominance of *α*-tocopherol [42–44].

In Morocco, limited studies have been conducted to determine the tocopherols profile of the same varieties cultivated in the country. Kodad et al. [45] have found approximate results for total tocopherols along with Mr: 401.6, Fn: 501.6, Fg: 461.9, and Fd: 401.1 mg/kg. In the literature available on tocopherols in almond oil, the variability in concentration and profile varies depending on the variety, the region, and the yearly crop [46, 47]. According to the EFSA (European Food Safety Authority), the average *α*-tocopherol absorption from a usual diet is about 75%, which is 13 mg/day for men, 11 mg/day for women, and 9 mg/day for children of both sexes aged 3 to <10 years and 6 mg/day if aged <3 years [48]. Vitamin E bioavailability is influenced by various factors, such as gender, age, and genotype, as well as environment factors, food habits, and lifestyle [49]. In terms of absorption, *α*-tocopherol is more assimilated by our body because *α*-TTP (alpha-tocopherol transfer protein) is a liver cytosolic transport protein that facilitates *α*-tocopherol (*α*-T) transfer. This later preferentially binds to *α*-tocopherol rather than other tocopherols or tocotrienols. Furthermore, the *ω*-hydroxylase, considered a key enzyme in the liver, has a much higher affinity towards *α*-tocopherol than other tocopherols [50]. Therefore, almond oil represents an important source of tocopherols, mainly *α*-tocopherol.

Table 3 shows the content of *α*-tocopherol of these four studied varieties planted in Spain, Turkey, and Argentina. This comparison could be useful for determining the effect of environmental conditions on the composition of almond oil between eastern Morocco and these countries. Our results for *Marcona* and *Fournat* are similar to those found in Spain (470 mg/kg), while the highest rate (595 mg/kg) is recorded for *Marcona* in Argentina. The value obtained for *Ferragnes* in eastern Morocco (401.23 mg/kg) was low compared to the value found in Spain (475 mg/kg), but remains higher compared to value found in Turkey (186.6 mg/kg), while the *Ferraduel* value (448.83 mg/kg) was higher compared to the value found in Spain (303 mg/kg). These recorded results confirm the geographical area effect on the composition of tocopherol. Differences between different work on different locations could be explained by the temperature variations and other environmental factors such as the rainfall and additional irrigation during fruit growing and ripening [45]. Moreover, the high values of our results encourage the plantation of these varieties in eastern Morocco and show good adaptability to the local conditions.

### 3.4. Fatty Acid Composition

The food authentication is a concern that has become more important over the past few years. The chemical composition such as fatty acid could be used as an efficient tool for adulteration detection and for determining the quality and the oil classification [56, 57]. Almond oil's fatty acid content is important in the diet. Due to a high proportion of unsaturated (MUFAs and PUFAs) fatty acids, the lipid fraction of almonds does not contribute to cholesterol production in humans. In most almond samples, oleic, linoleic, palmitic, and stearic acids (in decreasing order) account for approximately 95% of total FA concentration, while other FAs represent only 5%. The most abundant unsaturated fatty acids in almond oil are oleic and linoleic acids (approximately 90%), whereas saturated fatty acids, particularly palmitic, palmitoleic, and stearic acids, are quite low in quantity [58].  Table 4 shows the fatty acid composition of the studied varieties, where significant differences were observed between the varieties except for C17 : 0 (margaric acid). Profile of fatty acids in almond oil shows three major fatty acids: C18 : 1 (oleic acid) varied from 56.64% to 64.03% for Fn and Mr, respectively, C18 : 2 (linoleic acid) varied from 24.57% to 29.8% for Mr and Fn, respectively, and C16 : 0 (palmitic acid) varied from 7.22% to 8.60% for Mr and Fn, respectively. In addition, the analysis shows the presence of four minor fatty acids: C17 : 1 (margaroleic) varied from 0.076% to 0.093% for Fn and Fd, respectively, C17 : 0 (margaric acid) varied from 0.06% to 0.07% without any significant difference between the varieties, C16 : 1 (palmitoleic acid) varied from 0.56% to 0.74% for Fn and Fd, respectively, and C18 : 0 (Stearic acid) varied from 2.45% to 3.57% for Fd and Fn. The fatty acid profiles of almond oils show a low percentage of saturated fatty acids (SFAs) ranged from 10.13% to 12.24% which is nutritionally undesirable except for C18 : 0 that it is considered as less hypercholesterolemic because it is extensively converted to oleic acid [59].Unsaturated fatty acid (UFA) constitutes monounsaturated fatty acids (MUFAs) and polyunsaturated fatty acids (PUFAs).

The proportion of MUFA content shows significant differences between varieties. It varies from 57.28% to 64.85% for Fn and Mr, respectively, and the PUFA varied from 24.57% to 29.80% for Mr and Fn, respectively. UFA and C18 : 0 are desirable fatty acids (DFAs) with an average varied from 87.08% for Fn to 89.63% for Fd. Several studies referred to the benefits of MUFA intake on the risk factors of cardiovascular disease and the blood lipid profiles. Furthermore, the consumption of PUFA has demonstrated physiological benefits on heart rate, triglycerides, blood pressure, endothelial function, and cardiac diastolic functions [60, 61]. Overall, almond oils contain significant proportions of medicinal and nutritional desirable fatty acids with high ratios of UFA/SFA, where the higher value was recorded in Mr (8.82) and the lower in Fn (7.11); intermediate values were recorded for Fg and Fd. The World Health Organization (WHO) recommended the intake of SFA below 10 %E (Energy), MUFA with an average of 15 to 20 %E, and PUFA 6 to 11 %E according to total fat intake [62].

The ratio O/L ranged for 1.90 to 2.60 for Fn and Mr, respectively. This parameter can be employed to characterize almond oils and kernel cultivars [63] and also the stability of oil. The high variations in fatty acid compositions observed depend essentially on the difference between the genotypes. In addition, we observe a lower value of C18 : 1 and a high percentage of C18 : 2 and C16 : 0, if we compared oil extracted from the *Marcona* variety planted in eastern Morocco with its original site in Spain (Table 5). The same observation was obtained with *Ferragnes* and *Ferraduel* varieties in other sites, such as Argentina and Turkey. Only one study in Tunisia evaluated the composition of the *Fournat* variety shows a value less than our results [64]. However, other analyses carried out in our laboratory over 5 years show seasonal fluctuations in oleic and linoleic acid levels. Those observed variations in fatty acid compositions are probably related to several factors such as the growing region, crop year, climatic conditions during the growing season, and/or interactions of all these factors [39, 47].

### 3.5. Triacylglycerol Composition

Although triglycerides represent more than 98% of total lipids in almond oil [66], few studies have been conducted to determine the composition of triglyceride molecular species. Previous results have been able to identify nine molecular species and have shown varietal differences [65, 67]. The analysis of triacylglycerols (TAGs) in four studied almond oil varieties allows identifying eleven molecular TAG species. The three main TAGs, which constitute the most representative TAGs (72%) in the almond oil, are triolein (OOO) varied from 21.49% to 30% for Fn and Fd, respectively, dioleolinolein (OOL) ranged from 24.66 to 27.25% for Fg and Mr, respectively, and (LLO) ranged from 14.08 to 18.43% for Fg and Fn, respectively. The secondary TAG species identified are (LLS + POL), POO, LLL, LPL, SOO, POP, PPL, and SLO, where O = oleic, L = linoleic, P = palmitic, and S = stearic. The different proportions of TAGs are summarized in Table 6. Thus, the highest percentages of OOL in almond oils (24.66–27.25%) could present the authenticity indicator for the adulteration of almond oil compared to other oil, which are characterized by a less percentage of OOL, such as sunflower oil (OOL = 17.97 to 20.96%) [68] and a mixture of sesame and soybean oils (OOL = 6.73 to 19.46%) [69].

### 3.6. Oxidative Stability

The analysis of oxidative stability index (OSI) allows the prediction of almond oil shelf-life. The results in Table 1 showed significant differences between the four varieties. The lowest value of OSI was found in Fn with 17.25 h, while the highest value of OSI was recorded for Mr oil with 23.49 h. Intermediate values were recorded for Fg and Fd with 20.84 h and 20.20 h, respectively. The oxidative stability of almond oil was evaluated by other studies, at 100°C, where similar results were obtained with an average of 16.3 to 24.2 h [39, 70], while Kochhar [71] reported lower values of induction period 10.2 h in the same experimental conditions. The different values of induction periods are mainly related to the variation of the oil composition such as phenolic compounds, tocopherols, and fatty acids. Also, the stability of oil could be affected by processing and storage conditions, namely, the light, the temperature, the oxygen availability, and the transition metals [72, 73]. Furthermore, almond roasting previous to oil extraction increases the stability of almond oils [74]. The correlation coefficient (*r*) is statistical measurements that represent the proportion of the variance for a dependent variable, which is explained by independent variables in a linear regression model. Statistical data analysis (Table 7) between OSI and the several previously studied variables is conducted. The positive correlation was shown between the OSI and the O/L ratio (*r* = 0.95), content of phenols (*r* = 0.86), OOL (*r* = 0.78), and OOO (*r* = 0.68), while a negative correlation was found for C18 : 2 (*r* *=* −0.94), PUFA/SFA (*r* = −0.92), and LLL (*r* = −0.71). However, no correlation was obtained with tocopherol content. Thus, we concluded that almond oils of *Marcona*, *Ferraduel*, and *Ferragnes* are rich on phenols, have a high index of O/L, a less percentage of C18 : 2, and also characterized by a high OSI, while the almond oil of *Fournat* showed a less value of phenols, a high percentage of C18 : 2, and was characterized by the lowest OSI.

### 3.7. Principal Component Analysis

**Figure 1:**
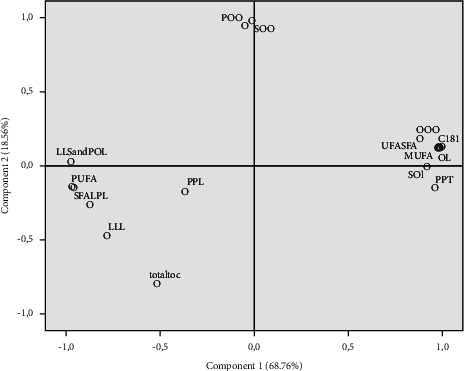
Projection of almond oil composition in the plane defined by the first two principal components.

**Figure 2:**
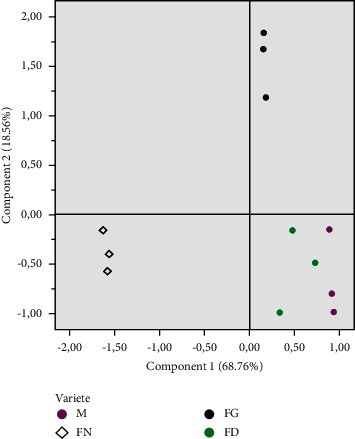
The dispersion of the four almond oils of the studied varieties in the plane defined by two principal components (M = *Marcona*, FN = *Fournat*, FG = *Ferragnes*, and FD = *Ferraduel*).

PCA is a technique for multivariate analyses that allows us to summarize and reduce the dimensionality of variables into main components. Although it is used to increase the interpretability, at the same time, it is used for minimizing the information loss. This analysis was used to explore and discriminate between the characteristics of each studied variety. The results of PCA are summarized in Figures 1 and 2. Two components were retained that explained 87.33% of the observed variability in the dataset. PC1 and PC2 accounted for 68.76% and 18.56%, respectively. The PC1 axis separated PPT, O/L, SOI, MUFA, UFA/SFA, C18 : 1, and OOO on the right side (Figure 2). On the opposite side, we found, especially, PUFA, LLL, LPL, (LLS + POL), and SFA. The PC2 is characterized by tocopherol in one side and POO and SOO in the opposite side. According to PC_S_ (PC1 and PC2), there are three mean groups: the first one is composed of Mr and Fd, which is characterized by a high stability of oxidation and richness of PPT and C18 : 1 and also a high value of O/L, while for the second group, it was composed only by Fn, characterized by a low stability of oxidation and a richness of PUFA (especially C18 : 2), SFA, LLL, and a low value of O/L. The third group is composed by the Fg variety related, especially, by PC2, which is characterized by a low value of tocopherol and a high value of POO and SOO. PCA analysis exposed clearly the variety effect on the composition and oil stability. Moreover, the varietal effect is even more evident when comparing Mr with Fn and Fg along with Fd, which are planted in the same orchard, and yet presents a different composition, especially for Mr and Fn.

## 4. Conclusions

In this study, the obtained results show significant differences among the composition of each variety. The profile of fatty acid presents high value of UFA and low value of SFA. The values of O/L index and PPT are the three major factors influencing the oil stability. A positive correlation was found between the oxidative stability and the high content of phenols and O/L index. The richness of tocopherol concentration in the four introduced varieties can be considered as an added value for the local production and to pursue new avenues with interesting nutritional and cosmetic properties of almond oil. The comparison of the zone effect between the international original sites of these varieties and its performance in the eastern region of Morocco shows the best suitability and adaptability of these varieties to our climate, manifested by the high value of minor compounds, especially the ones of phenols and tocopherols, as well as a good value of SOI. According to this study, *Marcona*, *Ferragnes*, and *Ferraduel* are three varieties recommended for new plantations in eastern Morocco.

## Figures and Tables

**Table 1:** Quality indexes of almond oils of the four studied varieties produced in eastern Morocco.

	*Marcona*	*Fournat*	*Ferragnes*	*Ferraduel*
Free acidity (% C18 : 1)	0.33 ± 0.04^a^	0.32 ± 0.04^a^	0.33 ± 0.02^a^	0.36 ± 0.03^a^
Peroxide value (meq O_2_ kg^−1^)	1.88 ± 0.60^a^	2.75 ± 0.25^b^	3.18 ± 0.36^c^	2.44 ± 0.28^b^
Oxidative stability (h) (OSI)	23.49 ± 0.50^c^	17.25 ± 0.25^a^	20.84 ± 0.09^b^	20.20 ± 0.20^b^

Significant differences in the same row are shown by different letters (a–c) for varieties (*p* < 0.05).

**Table 2:** Total phenol and tocopherol composition of four almond oil varieties produced in eastern Morocco.

	*Marcona*	*Fournat*	*Ferragnes*	*Ferraduel*
Total phenols (mg/kg)	141.66 ± 7.63^c^	85.33 ± 9.07^a^	116.91 ± 3.12^b^	133.64 ± 1.36^c^
*α*-tocopherol (mg/kg)	456.64 ± 2.93^b^	473.55 ± 4.77^c^	401.23 ± 3.82^a^	448.83 ± 7.59^b^
*β*-tocopherol (mg/kg)	1.67 ± 0.01^a^	3.43 ± 0.17^c^	2.43 ± 0.22^b^	2.11 ± 0.69^ab^
*γ*-tocopherol (mg/kg)	2.29 ± 0.01^a^	14.77 ± 0.51^d^	5.32 ± 0.06^b^	6.26 ± 0.14^c^
Total tocopherol (mg/kg)	460.61 ± 2.93^b^	491.77 ± 4.42^c^	408.99 ± 3.75^a^	457.21 ± 7.94^b^

Significant differences in the same row are shown by different letters (a–c) for varieties (*p* < 0.05).

**Table 3:** *α*-tocopherol content in almond oil of four European varieties planted in eastern Morocco compared to that in Spain, Turkey, and Argentina.

	Varieties					
	*Marcona*		*Fournat*	*Ferragnes*		*Ferraduel*
	Spain	Argentina	Spain	Turkey	Spain	Spain
*α*-tocopherol (mg/kg)	470.26^A^	595^B^	470^C^	168.6^D^	475^E^	303^F^
	**456.64** ^**∗**^		**473.55** ^**∗**^	**401.23** ^**∗**^		**448.83** ^**∗**^

^A^[51], ^B^[52], ^C^[45], ^D^[53], ^E^[54], ^F^[55]. ^*∗*^Value of *α*-tocopherol in eastern Morocco. Bold values indicate the results of this study.

**Table 4:** Fatty acid compositions of four almond oil varieties produced in eastern Morocco.

Fatty acids (%)	*Marcona*	*Fournat*	*Ferragnes*	*Ferraduel*
C16 : 0	7.22 ± 0.02^a^	08.60 ± 0.04^d^	7.32 ± 0.05^b^	07.71 ± 0.01^c^
C16 : 1	0.71 ± 0.01^b^	0.56 ± 0.05^a^	0.67 ± 0.06^ab^	0.74 ± 0.06^b^
C17 : 0	0.06 ± 0.00^a^	0.07 ± 0.01^a^	0.06 ± 0.01^a^	0.06 ± 0.01^a^
C17 : 1	0.09 ± 0.01^ab^	0.07 ± 0.00^a^	0.09 ± 0.01^ab^	0.09 ± 0.01^b^
C18 : 0	02.85 ± 0.02^b^	03.57 ± 0.06^c^	02.96 ± 0.02^b^	02.45 ± 0.05^a^
C18 : 1	64.03 ± 0.24^c^	56.64 ± 0.20^a^	62.68 ± 0.19^b^	62.69 ± 0.07^b^
C18 : 2	24.57 ± 0.06^a^	29.80 ± 0.17^c^	25.62 ± 0.10^b^	26.10 ± 0.35^b^
SFA	10.13 ± 0.05^a^	12.24 ± 0.11^b^	10.35 ± 0.08^a^	10.23 ± 0.07^a^
MUFA	64.85 ± 0.25^c^	57.28 ± 0.26^a^	63.44 ± 0.20^b^	63.53 ± 0.14^b^
PUFA	24.57 ± 0.10^a^	29.80 ± 0.17^c^	25.62 ± 0.10^b^	26.10 ± 0.35^b^
UFA	89.42 ± 0.31^b^	87.08 ± 0.43^a^	89.07 ± 0.28^b^	89.63 ± 0.49^b^
UFA/SFA	08.82 ± 0.01^c^	07.11 ± 0.03^a^	08.60 ± 0.07^b^	08.75 ± 0.04^c^
O/L	02.60 ± 0.10^d^	01.90 ± 0.15^a^	02.44 ± 0.19^c^	02.40 ± 0.14^b^
MUFA/SFA	06.39 ± 0.10^c^	04.67 ± 0.10^a^	06.12 ± 0.20^b^	06.20 ± 0.20^b^
PUFA/SFA	0.37 ± 0.00^a^	0.52 ± 0.01^d^	0.40 ± 0.10^b^	0.41 ± 0.10^c^

Significant differences in the same row are shown by different letters (a–d) for varieties (*p* < 0.05) (SFA: saturated fatty acid, MUFA: monounsaturated fatty acid, PUFA= polyunsaturated fatty acids, O: oleic, L: linoleic).

**Table 5:** Major fatty acids in almond oil of three European varieties planted in eastern Morocco compared to Spain, Turkey, Argentina, and Tunisia.

Major fatty acids (%)	Varieties					
	*Marcona*		*Ferragnes*		*Ferraduel*	*Fournat*
	Spain^AB^	Argentina^C^	Turkey^D^	Spain^B^	Spain^B^	Tunisia^E^
C16 : 0	6–6.36	6.22–6.55	6.87	6.49	6.64	6.84
	**7.22** ^**∗**^		**7.32** ^**∗**^		**7.71** ^**∗**^	**08.60** ^**∗**^

C18 : 1	67.90–74.6	70.1–71.5	74.63	71.81	67.52	69.56
	**64.03** ^**∗**^		**62.68** ^**∗**^		**62.69** ^**∗**^	**56.64** ^**∗**^

C18 : 2	16.4–19.20	19.7–21.4	19.51	17.62	21.47	20.77
	**24.57** ^**∗**^		**25.62** ^**∗**^		**26.10** ^**∗**^	**29.80** ^**∗**^

^A^[51]; ^B^[65]; ^C^[52]; ^D^[53]; ^E^[64].  ^*∗*^Value of fatty acids in eastern Morocco. Bold values indicate the results of this study.

**Table 6:** Triacylglycerol molecular species of four almond oil varieties produced in eastern Morocco.

Triacylglycerols (%)	*Marcona*	*Fournat*	*Ferragnes*	*Ferraduel*
LLL	03.24 ± 0.56^a^	05.18 ± 0.28^b^	03.06 ± 0.75^a^	3.65 ± 0.87^ab^
LLO	16.19 ± 2.02^a^	18.43 ± 0.04^a^	14.08 ± 1.51^a^	15.90 ± 2.21^a^
LPL	02.78 ± 0.11^a^	03.86 ± 0.64^b^	0.75 ± 0.34^a^	2.71 ± 0.46^a^
OOL	27.25 ± 0.49^c^	24.72 ± 0.19^a^	24.66 ± 0.19^a^	25.79 ± 0.24^b^
LLS + POL	09.27 ± 0.17^a^	12.32 ± 0.05^b^	10.21 ± 0.05^a^	9.63 ± 0.76^a^
PPL	0.34 ± 0.17^a^	0.42 ± 0.13^a^	0.3 ± 0.01^a^	0.33 ± 0.12^a^
OOO	28.72 ± 1.77^b^	21.49 ± 0.2^a^	28.12 ± 0.72^b^	30.00 ± 3.45^b^
POO	09.02 ± 1.03^a^	9.38 ± 0.15^a^	11.19 ± 0.73^b^	09.01 ± 0.46^a^
SLO	0.05 ± 0.00^a^	0.96 ± 0.26^b^	0.81 ± 0.53^ab^	0.12 ± 0.07^b^
POP	0.92 ± 0.08^a^	0.79 ± 0.18^a^	0.69 ± 0.09^a^	0.80 ± 0.05^a^
SOO	02.10 ± 0.22^a^	02.25 ± 0.16^a^	03.76 ± 0.24^b^	1.99 ± 0.27^a^

O = oleic, L = linoleic, P = palmitic, and S = stearic. Significant differences in the same row are shown by different letters (a–c) for varieties (*p* < 0.05).

**Table 7:** Coefficient of correlation (*r*) and variation between the oxidative stability of almond oil and O/L, C18 : 1, PPT, PUFA/SFA, C18 : 2, OOL, LLL, OOO, and *α*-tocopherol.

	*r*	*p*
O/L	0.95	<0.001
C18 : 1	0.92	<0.001
PPT	0.86	<0.001
PUFA/SFA	−0.92	<0.001
C18 : 2	−0.94	<0.001
OOL	0.78	<0.01
LLL	−0.71	<0.01
OOO	0.68	<0.05
*α*-tocopherol	0.30	NS

O: oleic, L: linoleic, PPT: total phenol content, PUFA: polyunsaturated fatty acids, SFA: saturated fatty acids, NS: no significance.

## Data Availability

All data generated or analyzed during this study are included within this article.
